# New Technology and Loss of Paid Employment among Older Workers: Prospective Cohort Study

**DOI:** 10.3390/ijerph19127168

**Published:** 2022-06-11

**Authors:** Emil Sundstrup, Annette Meng, Jeppe Z. N. Ajslev, Karen Albertsen, Flemming Pedersen, Lars L. Andersen

**Affiliations:** 1 National Research Centre for the Working Environment, 2100 Copenhagen, Denmark; ame@nfa.dk (A.M.); jza@nfa.dk (J.Z.N.A.); lla@nfa.dk (L.L.A.); 2 Team Working Life, 2500 Valby, Denmark; kal@teamarbejdsliv.dk (K.A.); flemp@teamarbejdsliv.dk (F.P.)

**Keywords:** technological change, digitalization, automation, health, retirement, senior workers

## Abstract

Background: This study investigates the association between the implementation of new technology in the workplace and the subsequent loss of paid employment among older workers. Methods: We estimated the prospective risk of loss of paid employment (register-based) from questions on new technology among 10,320 older workers (≥50 years). To investigate potential differences between work types, analyses were stratified by job function: (1) work with symbols (office, administration, analysis, IT), (2) work with people (people, service, care), (3) work in the field of production (processing, producing or moving things). Results: The introduction of new technology at the workplace reduced the risk of losing paid employment among older workers working with symbols (risk ratio [RR] 0.74, 95% CI 0.72–0.76) and in the field of production (RR 0.83, 95% CI 0.80–0.85), whereas new technology increased this risk among those working with people (RR 1.22, 95% CI 1.19–1.26). Being involved in the introduction of new technology and receiving adequate training in its use decreased the risk of loss of paid employment. Conclusions: Depending on the context, the introduction of new technology at work associates positively as well as negatively with future labour market participation among older workers. Worker involvement and adequate training in the use of new technology seem to be important for retaining workers in the labour market.

## 1. Introduction

Demographic changes in many industrialized countries reflect a growing proportion of older people. Consequently, many governments across the European Union are increasing the statutory retirement age and making it more difficult to retire early from the labour market. Along with demographic change, the labour market and the way we work have undergone fundamental changes in recent years. In the literature on the future of work, there is a growing consensus regarding technological change being the main driver—or megatrend—that shapes the working life of the future [1,2]. The technological change includes rapid progress in areas, such as computing, robotics, artificial intelligence, and biotechnology and applies, i.e., an increasing degree of automation and digitization, where the implementation of new technologies to a large extent—and across industries—changes the content of work, skill requirements and often also the organization of work. From a retention perspective, this may mean that the working population, including older employees, must show an even higher degree of adaptability to be able to handle a working life of the future and its challenges. At the same time, new technologies also have the potential for older workers to realize a long, healthy, and meaningful working life.

The technological change could be particularly challenging because older employees are assessed more negatively than younger workers in terms of mastering new technology and the willingness to learn new things [3,4]. Recent research into older workers’ labour market participation indicates that a stereotyping of older employees has an impact on the desire for retirement: negative stereotyping, e.g., management’s expectations that older employees will resist change, new technologies, competence development, and new knowledge, has been associated with early retirement [5,6]. Further, some studies are showing that older workers perceive the consequences of technological changes more negatively than their younger coworkers [7,8], while other studies do not [9]. In line with this, studies have shown that it is more difficult for older adults to handle technologies, even though they find them useful [10,11]. Older workers could, therefore, experience more situations with technologically-related stress arising from dependency on, e.g., information and communication technologies at work, which could induce stress and exhaustion [11,12]. Furthermore, a recent report employing data from Finland revealed that an individual aged 50 or above in occupations that are more exposed to digital technologies has a higher probability of exiting employment each year [13].

Age has also been shown to correlate negatively with technology acceptance. Implementation of new technology often fails because of worker resistance [14,15] that may occur due to distrust, fear of job loss and the feeling of being controlled [14,16,17]. Involvement of older workers in both planning, development, design, and testing of new technology can, therefore, be crucial for whether older workers are ready and willing to accept and use new technology [18]. Lack of technical and digital skills and the need for adequate training is one of the most significant obstacles to good implementation [14]; however, importantly, older adults’ digital literacy can be developed through exposure and education [19]. In line with this, Messe et al. investigated retirement intentions in the presence of technological changes and found that older workers who benefit from a skill upgrading training program have a higher intended retirement age [20].

Older workers are a highly differentiated group of employees, with diverse educational backgrounds and health, who perform many different types of work in very different work environments [21,22]. The technological solutions in the work, and how they affect labour market participation, could therefore be dependent on what kind of work the older employees perform. For example, the introduction of technology in production work can reduce the physical loading at work [23], and thereby help reduce the risk of a premature exit from the labour market [24,25], and at the same time, potentially create more motivating work tasks, where the planning and control of machines and robots become core competencies [26,27]. On the other hand, technology can also mean that many of the manual jobs found in the field of production are made redundant, whereby especially the least adaptable older workers can be pushed out of the labour market [28] if they are not adequately prepared for the technological development [1,29].

Although the implementation of new technology within health care work (working with people) also has the potential to reduce the physical loading, it may have other effects on push and stay than the introduction of technology in production work. Previous studies have shown that welfare technology in eldercare can be experienced as conflicts with the employees’ professional identity and values [27,30], which can potentially impair the quality of work and act negatively in the psychosocial and physical work environments [31]. Among older employees working with symbols (e.g., office work, research, and IT), a technological change may introduce more autonomy and flexible working environments and could facilitate a better work–life balance, while increased stress levels may arise from unlimited connectedness and blurred boundaries between work and private life [1]. Artificial intelligence will increasingly solve more of the data- and information-driven work tasks when working with symbols, which can change the content and organization of work. This can mean more time for complicated work tasks, an acceleration of work pace, and increasing job insecurity among the least adaptable.

New technology may, therefore, have different meanings for labour market participation, depending on what work the older workers perform. Although previous research suggests that there could be differences between job functions, this has not yet been investigated in larger studies that systematically compare job types. Furthermore, most research on older workers and technology has assessed participants’ intentions for future work participation rather than their actual behaviour. To our knowledge, no large scale longitudinal studies have employed survey and register information to assess the association between new technology and labour market participation among older workers, and no previous study has focused on this, concerning the specific job function of the older workers. Such knowledge could help identify vulnerable groups of older workers and initiate preventive efforts in the workplace to better unleash the potential of new technology as a contributor to a long, healthy and productive working life.

This study aims to investigate the association between the implementation of new technology at the workplace and register-based loss of paid employment among more than 10,000 older workers. To investigate potential differences between work types, the study has recruited employees across industries, which allows for stratification into three distinct job function categories: work with symbols, work with people and work in the field of production [32]. Hence, this study will answer the following research questions: Is the implementation of new technology at work associated with a loss of paid employment among older workers? Does this association differ according to older workers’ job functions?

## 2. Materials and Methods

### 2.1. Study Design

The study is based on the employee survey in the SeniorWorkingLife study, which is registered as a cohort study at ClinicalTrials.gov (identification number: NCT03634410, accessed on 8 June 2022). The baseline data collection took place between July and October 2018, where the respondents answered questions about, e.g., new technology in working life and further education and retraining [32]. A total of 18,000 employed Danes ≥ 50 years were invited to participate in the survey and received a personal questionnaire link in e-Boks (an online digital mailbox linked to the Danish social security number). For the analyses in this article, we included only currently employed wage earners.

Employed wage earners were defined based on three previously described criteria [32]. First, the person should have been in paid employment for at least 20/37 h per week (~86.6/160.3 h per month) for at least half of the months during the last year, as of March 2018. Second, the person should be employed at least 20/37 h per week during March 2018. As the last of the criteria, the person must not have received benefits from ‘flexible jobs’ (a job offer on special terms for people with permanently reduced work ability), “light jobs” (work on special terms with a wage subsidy offered to people on a disability pension), sickness absence, or maternity/paternity leave during the first quarter of 2018.

Among the invited employees, 56% answered all the survey questions (i.e., those who also answered other questions about the working environment and health, which are not included in this paper). The participants who only partially answered the survey questions (i.e., the questions used for this article and described below) were also included in the analyses, yielding a total study sample of 10,320 employed older workers. The characteristics of the entire study sample and stratified by job function category are presented in Table 1.

### 2.2. Subgroups of the Study Population

The following question was used to determine the job function category: “What do you work with first and foremost in your daily work?” with the following three response categories: (1) office work, administration, analysis, IT; (2) work with people, service, care; (3) work with processing, producing or moving things. For further analysis, the respondents were stratified in relation to these response categories: response category 1 was designated as work with symbols; response category 2 was designated as work with people; response category 3 was designated as work in the field of production [32,33]. In total, 8757 participants were included in the analysis (4364 working with symbols, 2872 working with people, 1521 working with production) whereas 1563 participants could not be categorized into the three job function categories.

### 2.3. Predictors

The following question initiated the questionnaire section about new technology in the working life: “Has new technology been introduced into your work within the last 2 years?” with the following response categories: “Yes”, “No” [33].

Those who answered “Yes” to the above question received the following five questions, with the introduction—“Do you agree with the following statements?”: (1) “The new technology can mean that I leave the labour market before state pension age?”, (2) “The new technology can mean that I stay at the labour market until after state pension age?”, (3) “I have been involved in the way the new technology has been introduced”, (4) “I need new skills as a result of the new technology ”, (5)“ I am offered adequate training in the use of the new technology ”. The response categories were: “Yes”, “No” and “Do not know”. For further analysis, we included only respondents who answered either “Yes” or “No”, and in the statistical analysis, respondents who indicated “No” to the individual question were used as a reference group [33].

### 2.4. Outcome

The outcome variable of ‘loss of paid employment’ from 2018 to 2020 was assessed from a national register on labour market affiliation, which was handled by Statistics Denmark. Loss of paid employment was defined as those who, in March 2020, no longer fulfilled the criteria of being employed (similar criteria as described in ‘Study Design’, but two years later). Importantly, the study population was truncated at the age of 63 at baseline, meaning that the included participants could not have left the labour market at or after the official state pension age during the follow-up period. In 2018, the state pension age was 65 years, but this was raised to 66 years in 2020. Thus, participants aged 63 years could not reach more than 65 years at follow-up, i.e., before state pension age. The outcome variable, therefore, defines the loss of paid employment before the state pension age.

### 2.5. Covariates

The statistical model was adjusted for the following potential confounders: age (years), gender (male/female), education (see below), body mass index (BMI: kg/m^2^), smoking status (No/Yes), and physical activity during leisure (see below).

Education was defined as the highest attained educational level and drawn from a national register handled by Statistics Denmark: (1) primary school or unknown, (2) high school, (3) short-term higher education, (4) medium-term higher education, and (5) long-term higher education [34].

Physical activity during leisure was assessed by the following question: “How would you describe your physical activity level during leisure for the last 12 months?”. Respondents were given the following response options (1) “Mostly sedentary”, (2) “Light exercise at least 4 h a week”, (3) “Sports or heavy physical activity at least 4 h per week” and (4) “Training and competing regularly and several times a week” [34,35].

### 2.6. Statistics

Using logistic regression (GLIMMIX procedure, SAS version 9.4, SAS institute, Cary, NC, USA) with model-assisted weights, representative risk ratios for loss of paid employment were modelled as a function of each predictor variable within the 2 year follow-up period. The statistical model was adjusted for age, gender, highest completed education, and lifestyle (BMI, smoking status, physical activity during leisure). The estimates are presented as risk ratios (RR) with 95% confidence intervals (CI).

## 3. Results

Table 1 illustrates the baseline characteristics of the study sample. Of the 10,320 older workers, the mean age was 55.8 years and 70% replied that new technology had been introduced into their work within the last two years. Of the study sample, 1755 older workers (weighted percentage = 12%) lost paid employment during the follow-up period from 2018 to 2020. Within each job function category, this was the case for 9.0% working with symbols, 13.4% working with people, and 13.7% working in the field of production. 

Table 2 and Table 3 illustrate the prospective association between loss of paid employment and the different aspects related to the introduction of new technology at work.

For the total study sample, the introduction of new technology at work was associated with a reduced risk of loss of paid employment (RR 0.86, 95% CI 0.85–0.87). When stratifying the participants based on job category, this was also observed for the groups working with symbols and in the field of production. On the contrary, among those working with people, the introduction of new technology increased the risk of loss of paid employment by 22% (RR 1.22, 95% CI 1.19–1.26).

Reporting that new technology can make you leave the labour market before the state pension age increased the risk of loss of paid employment by 53% (RR 1.53, 95% CI 1.49–1.56) whereas, stating that new technology can make you stay until after state pension age decreased the risk (RR 0.73, 95% CI 0.71–0.76). This picture was observed across all job categories.

Being involved in the introduction of new technology at work (RR 0.87, 95% CI 0.85–0.88) and being offered adequate training in the use of new technology at work (RR 0.58, 95% CI 0.57–0.59) was associated with a reduced risk of loss of paid employment. This was also observed across the three job categories. 

Needing new skills as a result of new technology at work was associated with an increased risk of loss of paid employment (RR 1.10, 95% CI 1.08–1.12). When stratifying on job category, this was also observed for those working with symbols and people. On the contrary, among those working in the field of production, needing new skills was associated with a reduced risk of loss of paid employment (RR 0.72, 95% CI 0.68–0.76).

## 4. Discussion

The introduction of new technology at the workplace reduced the risk of future loss of paid employment among older workers working with symbols and in the field of production, whereas new technology increased this risk among those working with people. For the entire study sample, reporting that new technology can prolong working life beyond the state pension age reduced the risk of loss of paid employment, whereas reporting that new technology can impact leaving prematurely increased this risk. Worker involvement in the introduction of new technology and adequate training in the use of new technology seems to be important for retaining older workers in the labour market (i.e., preventing future loss of paid employment). 

### 4.1. New Technology and Loss of Paid Employment

The majority of the older workers—across job function categories—stated that new technology had been introduced into their work within the past two years. However, a larger proportion of those working with symbols (75%) and people (71%) reported having new technology introduced compared to those working in the field of production (58%). Overall, the introduction of new technology in the workplace reduced the risk of future loss of paid employment among the study sample of older workers. However, when stratifying by job function, we observed clear differences across the three job function categories. While the introduction of new technology decreased the risk of future loss of paid employment among older workers working with symbols and in the field of production, it increased this risk among those working with people.

A previous study reported that, e.g., welfare technologies within work in elder care (work with people) can be experienced as being in conflict with the nursing staff’s professional identity and values [27]. This is supported by a qualitative study in the same industry, which describes that new technology, on the one hand, can reduce the physical strain at work and contribute to rehabilitation and self-help, while on the other hand, it can contribute to increased time pressure, reduced human interaction and job insecurity [31]. Whether these characteristics can be generalized across the employees who work with people—people, service, and care—cannot be answered by the present study. However, it may be hypothesized that the use of technology is more likely to conflict with the professional identity of workers working with people because the human interaction that takes place in working with people is so closely linked to the performance of core tasks. Several studies have also assessed the use of new technologies and how technology works together with professional identity among employees in the health care sector. Clark and Thompson showed how healthcare professionals experienced a reduction in meaning at work as a result of the introduction of new technology in journaling systems and in the standardization of the delivery of care tasks [36]. Other studies have also shown how professional identity in the health sector and elderly care is centred on values around care and nursing [37,38]. Although the fulfilment of these values may involve contradictions and conflicts in the interactions with the individual in need of care [39,40], this professional identity can contribute to the resistance of new technologies, either because they are perceived to get in the way of the caring aspects of work [36], or because they are perceived to reduce autonomy or professional discretion [41,42]. While the above-described considerations could be the best currently available explanations for the increased risk of future loss of paid employment among older employees working with people, the present study cannot establish a causal relationship between these things. However, it seems that the relation between new technology and loss of paid employment could be dependent on the type of work the older employees perform.

This also appears in line with the results on the relation between the intention of staying or leaving the labour market due to new technology and loss of paid employment. Workers who reported that new technology could mean that they would leave the labour market before the state pension age were at greater risk of a future loss of paid employment, whereas, those reporting that new technology could prolong their working life beyond the state pension age had a reduced risk of future loss of paid employment. However, those working with people experienced a higher risk of losing paid employment and a less reduced risk of staying compared to the two other job categories. Further, a larger proportion of older employees working with people replied, that new technology could impact their decision to leave prematurely (17% vs. 13% and 12%), whereas a smaller proportion replied that new technology can influence their decision to stay longer at work (12% vs. 22% and 22%). Overall, it seems that asking the workers if new technology can make them leave or stay can help identify vulnerable workers and initiate preventive efforts in the workplace.

### 4.2. Implementation of New Technology at Work

Involvement in the introduction of new technology at the workplace reduced the risk of loss of paid employment. This was observed across the three job function categories. The involvement of the older workers in the introduction of new technology, therefore, seems to be important for future labour market participation. Since about one-third of the older workers across job function categories reported not being involved in the introduction of new technology, there seems to be a great potential for companies to focus on this when implementing new technology in the future. For new technology to be able to contribute positively to the work, the user must be involved in the development process and the introduction [43]. As it is often the employees themselves who have the greatest knowledge and experience of their work, involvement in both planning, development, design, and testing of new technology can be crucial for whether the older workers are ready and willing to work with new technology [18]. If new technology is introduced engagingly and is perceived as meaningful, it may more easily find a place in the professional identity across job functions and help to raise the quality of work [30].

Furthermore, about 60% of the older workers needed new skills as a result of the introduction of new technology. We observed some differences across the job function categories, where a somewhat larger proportion of those working with people (63%) needed new skills compared to those working with symbols (58%) and in the field of production (49%). The statistical analyses showed that needing new skills increased the risk of loss of paid employment among those working with symbols and people, whereas it reduced this risk among those working in the field of production. It, therefore, seems that a lack of skills can push older employees working with symbols and people out of paid employment, whereas it acts as a stay factor for the older employees working in the field of production. It may be assumed, however, that both push and stay factors are involved when new technologies are requiring new skills from employees. On the one hand, the need for new competencies may act as a push factor for employees who do not want to or find it hard to gain these competencies, while on the other hand, it may be a stay factor for those who find it rewarding to learn new competencies and to be offered competence development within this area. It could be hypothesized, however, that the effect is to some degree dependent on whether the technology in question is perceived by the employee to improve the performance of central work processes or not. For employees working in the field of production, the use of technology is a central part of the processing, manufacturing, or transportation of materials, and calls for new skills may be more likely to be associated with better performance of central work processes and have the possibility to reduce physical workloads. For employees working with symbols, technology is often a medium for transportation or processing of the symbols, words, numbers, or pictures, but for many employees (e.g., teachers) is not by itself associated with better quality in the performance of central work processes. Often, new technologies in this area are more likely to improve administrative tasks. Therefore, the need for new competencies may not be as attractive for this group. Furthermore, particularly in the group of older employees working with people, the introduction of new technologies may often be connected with budget cuts, as this work is most often a part of core public services in which budgets have been reduced over the last decade or more [31]. Hence, older employees working with people may simply be correct in perceiving the implementation of new technologies as a threat to their continued employment. On the other hand, introducing technologies in production-oriented work may often be connected to a new investment or company growth and may, hence, mean increased opportunities rather than threats to employment. Indeed, studies have also pointed in the direction that implementation practices and a focus on securing return-on-investment of new technologies are at the centre of attention in private enterprises, which is not always the case in public service work [27].

Being offered adequate training in the use of new technology, as a part of the implementation, seems to be important for future labour market participation. Specifically, we found that adequate training reduced the risk of losing paid employment across all three job function categories, however, a significantly lower risk was observed among those working with symbols. Education and retraining—not necessarily related to new technologies—have previously been linked to the likelihood of remaining in the labour market [44,45,46], and could be a crucial instrument for increasing the older workers’ resources so that they can handle and operate new technology at work. A previous literature review of the factors that influence older nurses (working with people) to leave an organization or retire early identified career development and education as key factors that may influence this decision [47]. The authors argued that continuing education and skills development may be particularly important for older nurses (+50 years), as they have realized that the pace of technological change affects their ability to cope with their work [48]. In this context, it is also important to highlight the raw frequencies presented in Table 3, which show that those working with symbols (75%) are offered more adequate training in new technology compared to those working with people (59%) and in the field of production (63%).

### 4.3. Practical Applications

Work environment professionals and authorities should use this knowledge for early identification of workers at risk of a premature exit from the labour market. This knowledge can also be used when new technologies affect the majority of workers in a certain job group, e.g., the introduction of robots in nursing. Although the knowledge of the present study cannot stand alone, the development of practical guidelines on the implementation of new technology at work should build on the research. The present study provides updated knowledge on the prospective association between new technologies at work and the risk of losing paid employment. Such knowledge can be implemented in guidelines emphasizing, e.g., worker involvement in introducing new technologies, and education and training in its use. If new technology is introduced engagingly and is perceived as meaningful by the older workers, it may more easily find a place in professional identities across job functions and help to raise the quality of work. Given the importance of education for future labour market participation, it seems especially important that companies whose core task is working with people or in the field of production focus on designing and offering relevant training in the use of new technology. Especially if the new technology requires new skills and competencies. To better unleash the potential of new technology as a contributor to a long, healthy and productive working life, there should be a specific focus on worker involvement and adequate training and education in its use among older employees working with people.

### 4.4. Strengths and Limitations

Some strengths and limitations of the study need to be addressed. One strength is that Statistics Denmark drew a probability sample among all eligible Danish residents aged 50 years, which ensures (1) that everyone in the population (wage earners over the age of 50) can be selected, (2) that the selection is made by drawing lots, and (3) that the probability of being selected by drawing lots is known. As in all questionnaire surveys, the lack of responses may be a constraint on the representativeness of the survey. To take this into account, model-assisted weights were applied based on the following background variables: gender, age group, occupational industry, highest completed education, family income, family type, and origin. The weights were corrected using Statistics Denmark’s register’s information. Overall, the above procedures ensured that the data are representative of workers over the age of 50 in Denmark, which strengthens the generalizability of the study results [32,49].

Another strength of the study is the use of register-based data on the outcome variable: loss of paid employment before state pension age. The outcome variable was assessed from a national register handled by Statistics Denmark, containing daily information on wage and social transfer payments for all Danish residents.

A limitation to the study is that the stratification of job function (i.e., work with symbols, people, or in the field of production) was determined based on a question about what the older employees primarily work with. Hence, not all participants could be placed within the three distinct job function categories (i.e., replied working with “other things”), and 13% of the older workers were, therefore, not included in the stratified analyses. Even though we observed some clear differences between the job groups, this could have hampered the generalizability of the study results. A division based on register-based industry codes could have contributed to a more nuanced division and eliminated any bias associated with determining the job function based on the self-reports. On the other hand, within the same industry, people can have different work functions, e.g., in the construction industry, there are both office work and manual work.

Since the baseline measurements were assessed in 2018 and the follow-up period in the register was in 2020, just before the onset of the COVID-19 pandemic, the present study does not allow to estimate how the pandemic might have affected the association between the implementation of new technology and work participation. It is well known, however, that the COVID-19 crisis is having a drastic impact on new technology, work, and employment [50,51,52,53,54]. The pandemic-imposed lockdown has affected all industries and service sectors alike, including health care, manufacturing, and office work, among others, and has elevated the role of technology in the workplace [50,51,52,53,54]. For instance, technology has played a critical role in the healthcare sector (i.e., working with humans) due to the increased need for healthcare services during the pandemic [51], and has required a shift toward remote work for many information workers (i.e., working with symbols) [55]. These sudden changes have forced organizations and workplaces to adapt to the new conditions and accelerate new working and workplace models [53]. Future longitudinal studies should have a specific focus on the impact of technology among older workers during and following COVID-19 and how it associates with future labour market participation, and include stratified analyses on job function categories.

Another limitation is that the exposures (i.e., implementation of new technology at the workplace) were assessed by self-reports, which could have introduced reporting bias. Further, it was not possible based on the present study to go into detail with the type of new technology within each job function category. New technologies come in different forms and different technologies are introduced for different types of work. Hence, it seems plausible, then, that the relation between new technology and loss of paid employment may also depend on the type of technology being implemented. Future studies should, therefore, investigate the association between specific technologies and labour market participation. In addition, qualitative research is needed to better understand the relation between the types of new technology and loss of paid work, in particular, among those working with people.

## 5. Conclusions

The introduction of new technology at the workplace reduced the risk of future loss of paid employment among older workers working with symbols and in the field of production, whereas new technology increased this risk among those working with people. Worker involvement in the introduction of new technology and adequate training in the use of new technology seem to be important aspects of retaining older workers in the labour market.

## Figures and Tables

**Table 1:** Baseline characteristics of the entire study sample (N = 10,320) and stratified by job function category (work with symbols, work with people, work in the field of production).

	Study Sample		Symbols		People		Production	
	*N*	Percent or Mean (SE)	*N*	Percent or Mean (SE)	*N*	Percent or Mean (SE)	*N*	Percent or Mean (SE)
**Age, years**	10.320	55.8 (0.04)	4.364	55.4 (0.06)	2.872	56.2 (0.08)	1.521	55.6 (0.11)
**Gender**								
Women	4812	48%	2054	47%	2043	73%	265	16%
Men	5508	52%	2310	53%	829	27%	1256	84%
**Education**								
Unskilled manual worker	2043	21%	711	17%	420	15%	429	33%
Skilled manual worker	4389	41%	1675	38%	997	34%	952	59%
Further education	3888	38%	1978	45%	1455	51%	140	8%
**Smoking**								
No	8325	81%	3749	86%	2287	81%	1133	75%
Yes	1936	19%	590	14%	569	19%	380	25%
**BMI**								
<18	57	1%	20	0%	21	1%	7	0%
18–<25	4256	42%	1890	44%	1328	48%	488	31%
25–<30	4064	40%	1686	39%	1009	35%	701	47%
30–<35	1388	13%	550	13%	349	12%	243	16%
35–<40	321	3%	121	3%	86	3%	62	4%
≥40	103	1%	39	1%	35	1%	14	1%
**Physical activity during leisure**								
Seated	1494	14%	511	12%	386	13%	293	19%
Light exercise at least 4 h	6221	61%	2594	60%	1835	64%	891	59%
Sports or heavy physical activity at least 4 h per week	2358	23%	1135	26%	592	22%	310	20%
Training and competing regularly several times a week	203	2%	107	2%	45	2%	22	2%

**Table 2:** Association between new technology at work and loss of paid employment. RR = risk ratio, CI = confidence interval.

	*n*	Weighted %	RR (95% CI)
Has new technology been introduced into your work within the last 2 years?			
No	3331	30.1	1
Yes	6928	69.9	0.86 [0.85–0.87]
**New Technology and PUSH and STAY**			
The new technology can mean that I leave the labor market before state pension age? (PUSH)			
No	5189	85.8	1
Yes	881	14.2	1.53 [1.49–1.56]
The new technology can mean that I stay at the labour market until after state pension age? (STAY)			
No	4331	82.8	1
Yes	958	17.2	0.73 [0.71–0.76]
**Implementation of new technology**			
I have been involved in the way the new technology has been introduced?			
No	2405	35.4	1
Yes	4265	64.6	0.87 [0.85–0.88]
I need new skills as a result of the new technology?			
No	2658	40.8	1
Yes	3801	59.2	1.10 [1.08–1.12]
I am offered adequate training in the use of the new technology?			
No	2050	33.3	1
Yes	4141	66.7	0.58 [0.57–0.59]

Adjusted for age, gender, education, and lifestyle (BMI, smoking status, physical activity during leisure).

**Table 3:** Association between new technology at work and loss of paid employment stratified by job function category: work with symbols, work with people, work in the field of production. RR = risk ratio, CI = confidence interval.

	Symbols (*n* = 4364)		People (*n* = 2872)		Production (*n* = 1521)	
	%	RR (95% CI)	%	RR (95% CI)	%	RR (95% CI)
Has new technology been introduced into your work within the last 2 years?						
No	25.1	1	25.8	1	43.0	1
Yes	74.9	0.74 [0.72–0.76]	74.2	1.22 [1.19–1.26]	57.0	0.83 [0.80–0.85]
**New Technology and PUSH and STAY**						
The new technology can mean that I leave the labor market before state pension age? (PUSH)						
No	87.5	1	83.4	1	88.0	1
Yes	12.5	1.56 [1.50–1.63]	16.6	1.39 [1.35–1.43]	12.0	1.46 [1.38–1.55]
The new technology can mean that I stay at the labour market until after state pension age? (STAY)						
No	78.5	1	87.8	1	78.5	1
Yes	21.5	0.77 [0.73–0.81]	12.2	0.89 [0.85–0.94]	21.5	0.53 [0.48–0.58]
**Implementation of new technology**						
I have been involved in the way the new technology has been introduced?						
No	32.8	1	37.4	1	35.4	1
Yes	67.2	0.78 [0.75–0.80]	62.6	0.96 [0.93–0.98]	64.6	0.65 [0.62–0.68]
I need new skills as a result of the new technology?						
No	41.9	1	36.6	1	50.7	1
Yes	58.1	1.25 [1.21–1.29]	63.4	1.12 [1.09–1.16]	49.3	0.72 [0.68–0.76]
I am offered adequate training in the use of the new technology?						
No	25.3	1	40.7	1	37.5	1
Yes	74.7	0.58 [0.56–0.60]	59.3	0.68 [0.66–0.70]	62.5	0.65 [0.62–0.69]

Adjusted for age, gender, education, and lifestyle (BMI, smoking status, physical activity during leisure).

## Data Availability

The authors encourage the collaboration and use of the data by other researchers. Researchers who are interested in using the data for scientific purposes should contact the project leader, Lars L. Andersen, lla@nfa.dk.

## References

[1] Dølvik J.E., Steen J.R. (2018). The Nordic Future of Work.

[2] International Labour Office (2019). Work for a Brighter Future.

[3] McGregor J., Gray L. (2002). Stereotypes and Older Workers: The New Zealand Experience. Soc. Policy J. New Zealand.

[4] Meng A., Jensen P.H., Albertsen K., Sundstrup E., Andersen L.L. (2019). Stereotypier, Fordomme Og Aldersdiskrimination. Kapitel 12. Afrapportering af Projektet SeniorArbejdsLiv.

[5] Goul Andersen J., Jensen P.H. (2011). Tilbagetrækning fra Arbejdsmarkedet—Årsager og Effekter.

[6] Thorsen S.V., Jensen P.H., Bjørner J.B. (2016). Psychosocial Work Environment and Retirement Age: A Prospective Study of 1876 Senior Employees. Int. Arch. Occup. Environ. Health.

[7] Marquié J.C., Thon B., Baracat B. (1994). Age Influence on Attitudes of Office Workers Faced with New Computerized Technologies: A Questionnaire Analysis. Appl. Ergon..

[8] Tams S., Grover V., Thatcher J., Ahuja M. (2021). Grappling with Modern Technology: Interruptions Mediated by Mobile Devices Impact Older Workers Disproportionately. Inf. Syst. E-Bus. Manag..

[9] Berg-Beckhoff G., Nielsen G., Ladekjær Larsen E. (2017). Use of Information Communication Technology and Stress, Burnout, and Mental Health in Older, Middle-Aged, and Younger Workers—Results from a Systematic Review. Int. J. Occup. Environ. Health.

[10] Hauk N., Hüffmeier J., Krumm S. (2018). Ready to Be a Silver Surfer? A Meta-Analysis on the Relationship Between Chronological Age and Technology Acceptance. Comput. Hum. Behav..

[11] Hauk N., Göritz A.S., Krumm S. (2019). The Mediating Role of Coping Behavior on the Age-Technostress Relationship: A Longitudinal Multilevel Mediation Model. PLoS ONE.

[12] Gaudioso F., Turel O., Galimberti C. (2015). Explaining Work Exhaustion From a Coping Theory Perspective: Roles of Techno-Stressors and Technology-Specific Coping Strategies. Stud. Health Technol. Inform..

[13] Yashiro N., Kyyrä T., Hwang H., Tuomala J. (2020). Discussion Paper Series: Technology, Labour Market Institutions and Early Retirement: Evidence from Finland.

[14] Molino M., Cortese C.G., Ghislieri C. (2020). The Promotion of Technology Acceptance and Work Engagement in Industry 4.0: From Personal Resources to Information and Training. Int. J. Environ. Res. Public Health.

[15] Marler J.H., Dulebohn J.H. (2005). A Model of Employee Self-Service Technology Acceptance. Res. Pers. Hum. Resour. Manag..

[16] Cascio W.F., Montealegre R. (2016). How Technology Is Changing Work and Organizations. Annu. Rev. Organ. Psychol. Organ. Behav..

[17] Frey C.B., Osborne M.A. (2017). The Future of Employment: How Susceptible Are Jobs to Computerisation?. Technol. Forecast. Soc. Change.

[18] Grothe H., Bruus P. (2015). Den Teknologiske Stepmodel.

[19] Ha J., Park H.K. (2020). Factors Affecting the Acceptability of Technology in Health Care Among Older Korean Adults with Multiple Chronic Conditions: A Cross-Sectional Study Adopting the Senior Technology Acceptance Model. Clin. Interv. Aging.

[20] Messe P.-J., Moreno-Galbis E., Wolff F.-C. (2014). Retirement Intentions in the Presence of Technological Change: Theory and Evidence from France. IZA J. Labor Econ..

[21] Sundstrup E., Meng A., Poulsen O.M., Dyreborg J., Hougaard K.S., Flyvhom M., Andersen L.L. (2018). Seniorers Arbejdsmiljø og Helbred—Analyse til Seniortænketanken for et Længere & Godt Seniorarbejdsliv.

[22] National Research Centre for the Working Environment Arbejdsmiljø Og Helbred i Danmark 2012–2020.

[23] Arvidsson I., Balogh I., Hansson G.-Å., Ohlsson K., Akesson I., Nordander C. (2012). Rationalization in Meat Cutting—Consequences on Physical Workload. Appl. Ergon..

[24] Andersen L.L., Fallentin N., Thorsen S.V., Holtermann A. (2016). Physical Workload and Risk of Long-Term Sickness Absence in the General Working Population and among Blue-Collar Workers: Prospective Cohort Study with Register Follow-Up. Occup. Environ. Med..

[25] Sundstrup E., Hansen Å.M., Mortensen E.L., Poulsen O.M., Clausen T., Rugulies R., Møller A., Andersen L.L. (2018). Retrospectively Assessed Physical Work Environment during Working Life and Risk of Sickness Absence and Labour Market Exit among Older Workers. Occup. Environ. Med..

[26] Hinrichsen L. (2010). Manufacturing Technology in the Danish Pig Slaughter Industry. Meat Sci..

[27] Ajslev J.Z., Sundstrup E., Johansen H.H., Sørensen K., Jensen K.A., Poulsen O.M. (2019). Arbejdsmiljø Mæssige Udfordringer Som Følge Af Automatisering Og Digitalisering. En Rapport Om Trends Og Tendenser Med Relevans for Fremtidens Arbejdsmarked.

[28] European Agency for Safety and Health at Work (2017). Key Trends and Drivers of Change in Information and Communication Technologies and Work Location: Foresight on New and Emerging Risks in OSH: Working Report.

[29] Arntz M., Gregory T., Zierahn U. (2016). The Risk of Automation for Jobs in OECD Countries: A Comparative Analysis.

[30] Ajslev Z.N.A., Johansen H.H., Poulsen O.M. (2017). Nye Teknologier i Ældreplejen—Plejecentres Implementering Af Velfærdsteknologier Og Teknologiernes Betydninger for Arbejdsmiljø Og Fagidentitet. https://nfa.dk/da/Forskning/Udgivelse?journalId=9714af55-31c4-454f-9aa3-836745e75010.

[31] Ajslev J.Z.N., Højbjerg H., Andersen M.F., Andersen L.L., Poulsen O.M. (2019). Occupational Identities and Physical Exertion in (Re)Configurations of New Technologies in Eldercare. Nord. J. Work. Life Stud..

[32] Andersen L.L., Sundstrup E. (2019). Study Protocol for SeniorWorkingLife—Push and Stay Mechanisms for Labour Market Participation among Older Workers. BMC Public Health.

[33] Sundstrup E., Ajslev J., Andersen L. (2020). Sammenhæng Mellem Ny Teknologi i Seniorarbejdslivet Og Beslutningen Om at Forlade Arbejdsmarkedet Før Eller Efter Folkepensionsalderen. Tidsskr. Arb..

[34] Skovlund S.V., Bláfoss R., Sundstrup E., Thomassen K., Andersen L.L. (2020). Joint Association of Physical Work Demands and Leg Pain Intensity for Work Limitations Due to Pain in Senior Workers: Cross-Sectional Study. BMC Public Health.

[35] Calatayud J., Jakobsen M.D., Sundstrup E., Casaña J., Andersen L.L. (2015). Dose-Response Association between Leisure Time Physical Activity and Work Ability: Cross-Sectional Study among 3000 Workers. Scand. J. Public Health.

[36] Clark I., Thompson A. (2015). Healthcare Assistants: Distributional Losses as a Consequence of NHS Modernisation?: Healthcare Assistants. New Technol. Work. Employ..

[37] Mueller F., Valsecchi R., Smith C., Gabe J., Elston M.A. (2008). ‘We Are Nurses, We Are Supposed to Care for People’: Professional Values among Nurses in NHS Direct Call Centres. New Technol. Work. Employ..

[38] Waerness K. (1984). The Rationality of Caring. Econ. Ind. Democr..

[39] Tronto J. (1993). Moral Boundaries: A Political Argument for an Ethic of Care.

[40] Tronto J., Hirschmann N.J., Di Stefano C. (2001). An Ethic of Care. Voices of Community Care: Ethics, Aging and Caring Practices.

[41] Berg M. (1997). Rationalizing Medical Work: Decision Support Techniques and Medical Practices.

[42] Boonstra A., Boddy D., Fischbacher M. (2004). The Limited Acceptance of an Electronic Prescription System by General Practitioners: Reasons and Practical Implications. New Technol. Work. Employ..

[43] Cooke F.L. (2002). The Important Role of the Maintenance Workforce in Technological Change: A Much Neglected Aspect. Hum. Relat..

[44] Lawless M., Buggy C.J., Codd M.B. (2015). Educational Influences on Early Retirement through Disability in Ireland. Occup. Med..

[45] Laaksonen M., Rantala J., Järnefelt N., Kannisto J. (2018). Educational Differences in Years of Working Life Lost Due to Disability Retirement. Eur. J. Public Health.

[46] Møberg R.J., Goul Andersen J., Jensen P.H. (2011). Kapitel 7 Betydning Af Efteruddannelse for Fastholdelse Af Ældre På Arbejdsmarkedet. Tilbagetrækning fra Arbejdsmarkedet—Årsager og Effekter.

[47] Moseley A., Jeffers L., Paterson J. (2008). The Retention of the Older Nursing Workforce: A Literature Review Exploring Factors That Influence the Retention and Turnover of Older Nurses. Contemp. Nurse.

[48] Andrews J., Manthorpe J., Watson R. (2005). Employment Transitions for Older Nurses: A Qualitative Study. J. Adv. Nurs..

[49] Andersen L.L., Jensen P.H., Sundstrup E. (2020). Barriers and Opportunities for Prolonging Working Life across Different Occupational Groups: The SeniorWorkingLife Study. Eur. J. Public Health.

[50] Hodder A. (2020). New Technology, Work and Employment in the Era of COVID-19: Reflecting on Legacies of Research. New Technol. Work. Employ..

[51] Herath T., Herath H.S.B. (2020). Coping with the New Normal Imposed by the COVID-19 Pandemic: Lessons for Technology Management and Governance. Inf. Syst. Manag..

[52] Winter E., Costello A., O’Brien M., Hickey G. (2021). Teachers’ Use of Technology and the Impact of Covid-19. Ir. Educ. Stud..

[53] De Lucas Ancillo A., del Val Núñez M.T., Gavrila S.G. (2021). Workplace Change within the COVID-19 Context: A Grounded Theory Approach. Econ. Res.-Ekon. Istraživanja.

[54] Kniffin K.M., Narayanan J., Anseel F., Antonakis J., Ashford S.P., Bakker A.B., Bamberger P., Bapuji H., Bhave D.P., Choi V.K. (2021). COVID-19 and the Workplace: Implications, Issues, and Insights for Future Research and Action. Am. Psychol..

[55] Yang L., Holtz D., Jaffe S., Suri S., Sinha S., Weston J., Joyce C., Shah N., Sherman K., Hecht B. (2022). The Effects of Remote Work on Collaboration among Information Workers. Nat. Hum. Behav..

[56] The National Committee on Health Research Ethics What to Notify?. https://www.nvk.dk/forsker/naar-du-anmelder/hvilke-projekter-skal-jeg-anmelde.

